# Serotonin Transporter Gene-Linked Polymorphism Affects Detection of Facial Expressions

**DOI:** 10.1371/journal.pone.0059074

**Published:** 2013-03-14

**Authors:** Ai Koizumi, Norimichi Kitagawa, Hirohito M. Kondo, Miho S. Kitamura, Takao Sato, Makio Kashino

**Affiliations:** 1 NTT Communication Science Laboratories, NTT Corporation, Kanagawa, Japan; 2 Department of Psychology, The University of Tokyo, Tokyo, Japan; 3 Core Research for Evolutional Science and Technology, Japan Science and Technology Agency, Kanagawa, Japan; 4 Research Center for Advanced Science and Technology, The University of Tokyo, Tokyo, Japan; 5 Interdisciplinary Graduate School of Science and Engineering, Tokyo Institute of Technology, Kanagawa, Japan; Radboud University, The Netherlands

## Abstract

Previous studies have demonstrated that the serotonin transporter gene-linked polymorphic region (5-HTTLPR) affects the recognition of facial expressions and attention to them. However, the relationship between 5-HTTLPR and the perceptual detection of others' facial expressions, the process which takes place prior to emotional labeling (i.e., recognition), is not clear. To examine whether the perceptual detection of emotional facial expressions is influenced by the allelic variation (short/long) of 5-HTTLPR, happy and sad facial expressions were presented at weak and mid intensities (25% and 50%). Ninety-eight participants, genotyped for 5-HTTLPR, judged whether emotion in images of faces was present. Participants with short alleles showed higher sensitivity (*d*′) to happy than to sad expressions, while participants with long allele(s) showed no such positivity advantage. This effect of 5-HTTLPR was found at different facial expression intensities among males and females. The results suggest that at the perceptual stage, a short allele enhances the processing of positive facial expressions rather than that of negative facial expressions.

## Introduction

Recent research has revealed that whether one prioritizes the processing of positive or negative information can be at least partially explained by our genetic composition. In particular, the allelic variation of the serotonin transporter gene-linked polymorphic region (5-HTTLPR) has been shown to affect emotional information processing among healthy individuals. The 5-HTTLPR has a low-functioning allelic variation (i.e., a short allele) and a high-functioning allelic variation (i.e., a long allele), and they exhibit hindered and enhanced transcriptional activity, respectively [1], [2]. More specifically, the 5-HTTLPR has short (S) and long (L) allelic variations, with a further variation of single nucleotide polymorphism, A or G, within the L-allele (La or Lg). The S allele has been shown to have lower transcriptional activity than the L allele [1], and more recently, Lg has been shown to have lower transcriptional activity than La, functioning similarly to the S allele [2]. While S- versus L-allele corresponds to low- and high- functioning allele in some studies [3], S- and Lg-allele versus La-allele corresponds to low- and high-functioning allele in others [4]. As studies have shown similar results regardless of which grouping method was used [5], to be more comprehensive, we simply use the terms low- and high-functioning alleles regardless of the grouping method used in each study when referring to past studies or making general arguments.

The accumulating evidence has revealed that the low-functioning allele typically enhances the processing of negative information over positive information, while the high-functioning allele enhances the processing of positive information. For example, individuals with a low-functioning allele show an attentional bias towards the location of negative facial expressions, resulting in faster responses to neutral probe stimuli presented afterwards at that location, and/or reduced attention towards the location of positive facial expressions [4], [6]. In contrast, individuals with a high-functioning 5-HTTLPR allele show enhanced attentional allocation towards the location at which positive facial expressions are presented [4].

An interesting question arises from these findings as to whether individuals with high- and low-functioning alleles differ in the way they perceive others' facial expressions. This is because facial expressions convey important social cues, and thus any bias in processing facial expressions should have a significant impact on daily social interactions. A recent seminal work examined this question as regards the recognition of facial expressions [7]. They measured the lowest intensity of facial expression at which participants could identify an expressed emotion, such as happy, sad, anger, and fear. Although their results were somewhat complicated, one of their main findings was that carriers of the low-functioning allele could generally recognize negative facial expressions at a lower intensity. A number of studies have examined the processing of facial expression using recognition tasks and have revealed various aspects of emotional information processing [8]. However, the facial expression recognition tasks used by Antypa et al. [7] and in many previous studies, involve two facial expression processing stages: perceptually detecting the facial expression and labeling it correctly with a certain emotion category [8], [9]. Therefore, we cannot assess whether the observed bias in facial expression recognition is due to a perceptual bias when detecting facial expressions or due to a response bias when labeling them to emotion categories.

The effects of 5-HTTLPR on the detection stage of facial expressions may be expected to differ from those observed in a previous study involving the labeling stage [7] because an act of labeling (i.e., explicitly linking the perceived emotional information with conceptual knowledge of emotion categories) has been consistently shown to regulate neuronal activity during the encoding of facial expressions [10]. Specifically, a task that consists of labeling facial expressions, compared with other types of tasks involving the encoding of facial expressions without labeling, has been shown to down-regulate amygdala activity towards facial expressions by enhancing the inhibitory control of the ventrolateral and medial prefrontal cortex [10]. As 5-HTTLPR has been shown to modulate the functional and anatomical coupling between the amygdala and prefrontal areas [11], [12], it is not surprising that 5-HTTLPR affects the facial expression labeling performance [7]. However, given that the mere perceptual encoding of facial expressions without labeling recruits prefrontal control only to a lesser extent [10], whether and how 5-HTTLPR would affect the detection of facial expressions without the involvement of labeling cannot be inferred from a previous study with a recognition task and thus requires direct examination. Decomposing the complex stages of facial expression processing and revealing the effects of 5-HTTLPR on a specific stage of facial expression processing (i.e., the detection stage) will contribute to our understanding of how 5-HTTLPR affects our emotional information processing.

In the present study, to eliminate the involvement of the response bias towards a specific emotion category in the labeling stage, we employed a detection paradigm for facial expressions in which participants were presented with facial expression images and required to judge the “presence” or “absence” of emotional expression in the face (i.e., the detection of facial expressions) irrespective of the emotion categories. We calculated the perceptual sensitivity (*d*′) to facial expressions, independent of the effect of the response bias towards the ‘present’ response using signal detection theory [13]. As previous studies undertaken with recognition tasks often report a higher recognition rate for happy facial expressions compared with other expressions [8], [14], we aim to examine whether this positivity advantage would be observed even at the perceptual detection stage. More importantly, we aim to examine whether such a positivity advantage would be observed differently for carriers of low-functioning alleles (i.e., a short allele) and carriers of high-functioning allele(s) (i.e., a long allele).

## Methods

### Ethics Statement

The experiments were conducted in accordance with the ethical standards of the 1964 Declaration of Helsinki and approved by the Research Ethics Committee of NTT Communication Science Laboratories. The participants provided written informed consent prior to their inclusion in the study.

The participants were 98 healthy Japanese (48 males, mean age 27 years, range 20–35 years). They were naïve as to the purpose of the study and were paid for their participation. The participants were genotyped for 5-HTTLPR (see [15] for genotyping methodology). With Asian participants, one should consider that while the L-allele (i.e., high-functioning allele) is dominant among those of African and European descent, the S-allele (i.e., low-functioning allele) is dominant among East Asians [16]. Given this difference in allelic variation, previous studies with Asians e.g., [17] have often contrasted S/S carriers (65%) with the S/L and L/L allele carriers (approximately 32% and 3%, respectively; cf. [18]). Following these studies, we divided the 98 Japanese participants into S/S-carriers versus S/L- or L/L-carriers (L-carriers). Although recent studies have considered a further variation of single nucleotide polymorphism A or G within the L-allele (La or Lg) due to lower transcriptional activity in Lg than La [2], the very low frequency of the L-allele in Japanese populations does not allow us to examine the variation in single nucleotide polymorphism. However, the present study can still contribute to revealing the effect of the 5-HTTLPR, as previous studies without such subtypes have elucidated the effects of the 5-HTTLPR [5]. Moreover, a few recent studies have suggested that Asians differ from those of European descent as regards the effects of 5-HTTLPR on amygdala activity [17], [19] and vulnerability to mood disorders [20]. As most of the studies that have examined the effects of 5-HTTLPR on emotion information processing were conducted with participants all or most of whom were of European descent [3], [4], [7], [21] and only a few studies have yet been conducted with Asian populations [15], [17] the current study will provide further data regarding the effects of 5-HTTLPR on emotional functions in the Asian population.

Since a number of studies have shown that females are generally more sensitive to subtle facial expressions than males [22], [23], we also consider the participants' gender. The four demographic groups arising from the combination of the 5-HTTLPR and gender were male S/S-carriers, female S/S-carriers, male L-carriers, and female L-carriers. The depression level [24] measured prior to the experimental session did not differ across the groups. The size and mean depression level of each demographic group are listed in Table 1. The groups did not differ in terms of mean age.

**Table 1:** Number of participants in each demographic group and their mean depression (Beck depression inventory, BDI) level.

	S-carriers		L-carriers	
Measure	Male	Female	Male	Female
n	31	35	17 (2)	15 (4)
Depression level (BDI)	8.4	11.9	9.1	9.7

*Note*. The numbers of L/L-carriers among L-carriers are given in parentheses.

**Figure 1:**
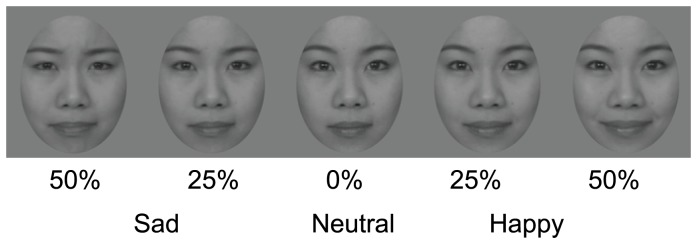
Examples of images of facial expressions at different intensities.

Facial images of four Japanese male and four Japanese female models expressing sad, happy, and neutral facial expressions were taken from a commercial database (ATR DB-99, http://www.atr-p.com/face-db.html). The models were trained to express the intended emotions with reference to the specific action units composing different facial expressions [25], and the developer confirmed the abilities of the facial expressions to convey the intended emotions. The sad and happy facial images of each model were morphed from a neutral facial expression of the same model. We used facial images at low (25%) and mid (50%) intensities of the sad and happy facial expressions as well as the neutral facial images (Figure 1). As a previous study showed that happy facial expressions are recognizable at a low intensity near 30% and that sad facial expressions are generally more difficult to recognize than happy facial expressions [8], happy and sad facial expressions at 25% intensity would be relatively difficult to detect, while the facial expressions at 50% would be easy for most individuals to detect. The facial images were converted into gray scale images, and cut into oval shapes (3.3°×4.3° in visual angle). The overall luminance of the images was matched. The experiment was controlled by MATLAB (Mathworks) on a PC (Apple, Powerbook G4) with the Psychophysics toolbox [26].

Each trial started with the presentation of a fixation cross at the center of a gray background. After a random period of 1 to 1.5 s, the fixation point disappeared and one of the facial images was presented for 200 ms. The participants were asked to make responses indicating whether the face expressed any emotion (“present”) or not (“absent”) irrespective of whether it expressed a sad or happy emotion. Responses were made by pressing a key. The participants' responses were followed by the next trial.

We presented two different types of emotion-present trials (i.e., happy and sad faces) with a set of emotion-absent trials (i.e., neutral faces) in the same single block, rather than in two separate blocks. This single block design was used to avoid creating a prior expectation as to which type of facial emotion (happy or sad) would be presented next, since such expectation could modulate the perception in a top-down manner [27]. The experimental session began with ten practice trials followed by a test block in which the 32 emotional faces (2 facial expressions ×2 intensities×8 models) were presented three times and the neutral faces from the eight models were presented six times to balance the numbers of sad, happy, and neutral facial expression trials, yielding 144 trials in total. The presentation order was randomized for each participant. A session lasted for approximately 10 min for each participant.

While the inclusion of two different signals (i.e., happy and sad faces) in a single block is effective in measuring perceptual sensitivity to facial expressions as discussed above, it has been shown that when multiple signals are presented in the same context, participants tend to adopt a single criterion (i.e., response bias) when making judgments (i.e., “present/absent” in the current case) rather than two separate criteria for two different signals [28]. Thus, in the current task, it is difficult to differentiate the participants' response bias towards the ‘present’ response between the happy and sad faces. Therefore, we will only focus on the sensitivity (*d*′) towards happy and sad faces and will not discuss the response biases.

The signal detection theory [29] was used to calculate sensitivity (*d*′), excluding the response bias. The *d*′ score was calculated for each participant and for each condition from the *hit rate* (i.e., the ratio of “present” responses to the emotional faces) and *false alarm rate* (i.e., the ratio of “present” responses to the neutral faces) as follows: *d*′ = Φ^−1^(*hit rate*)−Φ^−1^(*false alarm rate*), where Φ^−1^ (i.e., inverse phi) converts the probability scores into z-scores [13], [30]. Briefly, a higher *hit rate* and a lower *false alarm rate* would yield a larger *d*′ score (higher sensitivity to the emotional facial expressions).

## Results

The proportion of “present” responses to the facial-expression-present trials (i.e., *hit rate*) and that to the facial-expression-absent trials (i.e., *false alarm rate*) for each condition are shown in Table 2. To test whether the perceptual sensitivity to facial expressions, especially the superior sensitivity to the happy over sad (i.e., positivity advantage), was affected by 5-HTTLPR and Gender, the sensitivity scores (*d*′) were analyzed by a four-way analysis of variance (ANOVA) with two between-participants factors of 5-HTTLPR (S/S- vs. L-carriers) and Gender (male vs. female) and two within-participants factors of Facial expression (happy vs. sad) and Intensity (25% vs. 50%).

**Table 2:** The proportion of “present” responses towards the face images in each demographic group.

		S-carriers		L-carriers	
Emotion	Intensity	Male	Female	Male	Female
Happy	25%	56.12 (4.13)	60.06 (3.13)	50.98 (4.54)	51.94 (4.96)
	50%	90.66 (3.26)	92.68 (1.38)	85.54 (2.55)	91.53 (2.59)
Sad	25%	50.60 (3.79)	45.54 (3.61)	41.91 (5.48)	45.56 (5.31)
	50%	81.52 (3.59)	80.30 (4.01)	85.91 (2.82)	82.22 (4.64)
Neutral	0%	25.20 (2.90)	22.71 (2.67)	21.69 (3.21)	18.61 (3.13)

*Note*. Standard deviations are presented in parentheses. The proportions of “present” responses for happy and sad facial expressions indicate the *hit rate*, whereas those for neutral faces indicate the *false alarm rate*.

**Figure 2:**
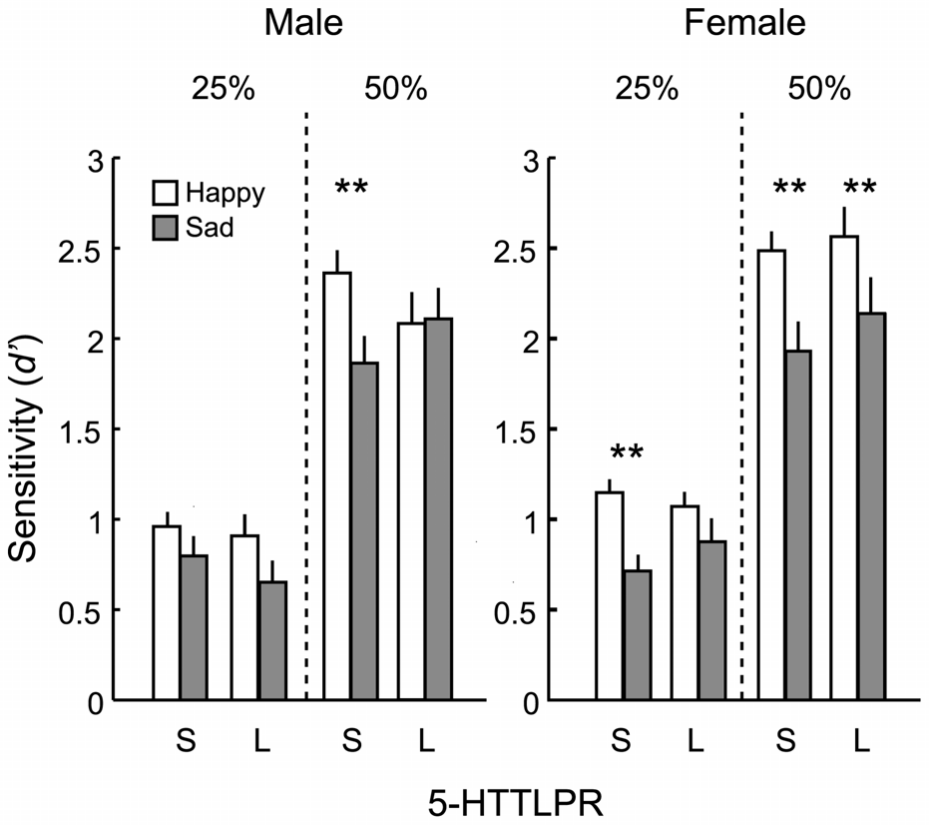
Mean sensitivity (*d*′) to happy and sad facial expressions at two intensities (25% and 50%). Means are shown separately for each demographic group among males (left) and among females (right). The error bars denote the standard errors of the means. The asterisks indicate the significance of the positivity advantage (i.e., significant difference in *d*′ between happy and sad facial expressions: **p<0.1).

The sensitivity scores were higher for the high intensity (50%) than for the low intensity (25%) facial expressions, resulting in a significant main effect of Intensity [*F*(1, 94) = 529.75, *p*<0.001, η_p_^2^ = 0.85]. The main effect of 5-HTTLPR was not significant [*F*(1, 94) = 0.03, *p* = 0.87]. Although the main effect of Gender was not significant, females (*M* = 1.62) showed numerically higher sensitivity to the facial expressions than males (*M* = 1.47). The main effect of Facial expression was significant [*F*(1, 94) = 32.22, *p*<0.001, η_p_^2^ = 0.26], with higher sensitivity to the happy than to the sad facial expressions, which agreed with the overall positivity advantage reported in previous studies involving recognition tasks [8], [14]. This positivity advantage depended on 5-HTTLPR, Gender, and Intensity, resulting in a significant three-way interaction between 5-HTTLPR, Facial expression, and Intensity [*F* (1, 94) = 4.04, *p* = 0.047, η_p_^2^ = 0.04], and more importantly, in a highly significant four-way interaction between 5-HTTLPR, Gender, Facial expression, and Intensity [*F*(1, 94) = 8.09, *p* = 0.05, η_p_^2^ = 0.8]. Post-hoc comparisons (Bonferroni correction) revealed that S/S-carriers (i.e., low-functioning allele carriers) showed an enhanced positivity advantage (i.e., significantly higher sensitivity for the happy faces than for the sad faces) compared with L-carriers (i.e., high-functioning allele carriers) both among males and females, but this group difference was observed with the facial emotions expressed at a higher intensity among males than females (see Figure 2). With the male participants, the positivity advantage was significant only when the S/S-carriers detected a facial expression of 50% intensity (*p*<0.001). In contrast, with the female participants, both the S/S- and L-carriers showed a significant positivity advantage for 50% intensity, but only S/S-carriers showed a significant positivity advantage for a subtle facial expression of 25% intensity (*p*<0.001).

We also conducted an analysis to test whether 5-HTTLPR affects the *false alarm rate*. This is because neutral faces could be subjectively perceived as emotionally cold and have been shown to be associated with a negative valence rather than neutrality [31]. However, in the current study, the *false alarm rate* was unaffected by 5-HTTLPR (*p* = 0.39), Gender (*p* = 0.37), and their interaction (*p* = 0.92). This suggests that 5-HTTLPR does not modulate the perception of neutral faces.

## Discussion

The present study examined whether the 5-HTTLPR affects the perceptual sensitivity to happy and sad facial expressions among healthy individuals. Previous studies have examined the influence of 5-HTTLPR on the recognition of facial expressions. However, a major limitation of recognition tasks is that they cannot differentiate between the detection of facial expressions and labeling them with an emotion category [8], [9]. The detection paradigm in the present study eliminated the contribution of the labeling stage and revealed the effect of 5-HTTLPR specifically on the detection of facial expressions.

The results revealed for the first time that the positivity advantage (i.e., positive facial expressions were identifiable at a lower intensity of facial expression than with negative facial expressions), which has been reported in facial expression recognition tasks in previous studies [8], [32], can be observed even in the detection task (mean sensitivity score (*d*′) of 1.71 and 1.36 for the happy and sad facial expressions, respectively). This suggests that the positivity advantage of the perception of facial expressions could arise at the detection stage. It would be interesting to examine the differences and similarities of the positivity bias between tasks without emotional labeling (i.e., the current study) and tasks with labeling [32] in future studies.

The results also revealed that the positivity advantage indexed as the difference in *d*′ between the happy and sad facial expressions was generally larger in the S/S-carriers (i.e., low-functioning alleles carriers) of the 5-HTTLPR compared with the L-carriers (i.e., high-functioning allele(s) carriers) (compare the difference between the white and gray bars in Figure 2). This group difference was observed at the different intensities of facial expressions for males and females. Thus, we revealed the effects of 5-HTTLPR on the perceptual detection of facial expressions eliminating the contribution of the labeling stage involved in recognition tasks. It is important to note that there was no difference in *false alarm rates* between the genetic groups, suggesting that the 5-HTTLPR does not affect the extent to which one detects emotions in neutral faces (i.e., *false alarm*). This implies that the above-mentioned results for the positivity advantage cannot be explained by the altered perception of neutral faces.

The enhanced positivity advantage among S/S-carriers observed in the present study appears to be different from the finding that the recognition of negative facial expressions, rather than positive ones, is enhanced among those carrying the low-functioning allele of the 5-HTTLPR [7]. Recognition tasks involve not only the detection of facial expressions but also the labeling of facial expressions with emotion categories [8], [9]. Verbal labeling of the perceived facial expressions with conceptual knowledge of emotion categories has been repeatedly shown to down-regulate the amygdala activity towards facial expressions by recruiting the inhibitory control of the prefrontal areas [10], suggesting that the verbal labeling of emotional information works as a means of emotion regulation [10]. Thus, the improved recognition of negative facial expressions among low-functioning allele carriers [7] could be due to the insufficient down-regulation of the amygdala activity towards negative facial expressions. This agrees with previous findings showing that low-functioning allele carriers exhibit reduced top-down regulation of the amygdala activity when processing negative facial expressions [3]. On the other hand, low-functioning alleles may enhance the processing of positive facial expressions at the perceptual detection stage where labeling is not required and thus down-regulation of the amygdala activity by the prefrontal areas would be less involved. As the mechanisms underlying the mere detection of facial expressions without labeling have not been directly tested, it would be worth examining such mechanisms as well as the neuronal mechanisms underlying the enhanced positivity advantage at the detection stage among low-functioning allele carriers.

It has been shown that when negative and neutral facial expressions are simultaneously presented, the carriers of the low-functioning allele show attentional bias towards the location of negative facial expressions [4], [6], [15]. In contrast, when facial expressions were presented along with other facial expressions but with less competing processing (i.e., free viewing with no time pressure or task demand to prioritize one facial expression over another), carriers of low-functioning alleles directed their gaze towards happy facial expressions for longer durations than towards negative facial expressions [33]. An explanation for these contrasting findings could be that the processing competition between negative facial expressions and other information recruits the regulatory control of prefrontal areas over amygdala activity [34]. The low-functioning allele carriers may show attentional bias towards negative facial expressions when competition between the processing of facial expressions and other information recruits the regulatory control of the amygdala, whereas they may show a bias towards positive facial expression processing in the absence of such competition. Future research should directly test whether the same neuronal mechanisms underlie the two sets of contrasting effects of 5-HTTLPR, i.e., those between the detection and categorization of facial expressions and those between the attentional allocation towards facial expressions with and without processing competition.

Another interesting explanation for the difference between the current finding and those of previous studies regarding whether low-functioning allele carriers show positive or negative bias in information processing would be that such a difference arises from the variance in the populations involved in the studies. Some recent studies have shown that the low-functioning alleles in Asian populations are linked with lower amygdala activity towards negative information [17] and in a resting state [19] as well as with a lower prevalence of mood disorders [20]. The results have often been linked with the high-functioning alleles rather than the low-functioning alleles in studies conducted with participants who were mostly or solely of European descent [21], [35]. It has been suggested that culture serves as a critical environmental factor that modulates the effects of 5-HTTLPR [20], which could explain why low-functioning, rather than high-functioning allele carriers, showed more positivity advantage in detecting facial expressions in the current study with Japanese participants. However, our previous study with Japanese participants did find that low-functioning allele carriers are more likely than high-function allele carriers to experience interference from negative information (i.e., negativity bias) when presented with two competing pieces of emotional information [15]. This agrees more closely with studies of those of European descent where there is a stronger negativity bias among low-functioning allele carriers than high-functioning allele carriers. One interesting possibility to test in future studies is that the higher sensitivity to positive facial expressions found here may counteract such a negativity bias in inhibitory control [15], resulting in higher resilience to mood disorders among Asian S-allele carriers [20].

We found an interaction between 5-HTTLPR, Gender, Facial expression, and Intensity resulting from the fact that the effect of 5-HTTLPR on the positivity advantage was different between males and females depending on the intensity of the facial expressions. Among the female participants, both S/S- and L-carriers showed a significant positivity advantage for the 50% obvious facial expressions, but S/S-carriers only showed a significant positivity advantage for the 25% weak facial expression. On the other hand, among the male participants, neither S/S- nor L-carriers showed a positivity advantage for the 25% weak facial expressions, while S/S-carriers only showed a positivity advantage for 50% obvious facial expressions. These results may be attributed to the gender difference in the baseline sensitivity to facial expressions: In previous studies, females have been shown to be generally more sensitive to subtle facial expressions than males [22], although this trend did not reach significant level in our experiment, possibly due to the fact that we used only limited levels of facial expression intensities. We can speculate that, considering this generally found gender difference in sensitivity to facial expressions, any individual differences between S- and L-carriers in females could be less likely to be observed when obvious facial expressions are presented because most females can detect emotions in those facial expressions. In contrast, differences between S- and L-carriers among males may be less likely to be observed when subtle facial expressions are presented because most males would find it difficult to detect emotions in them.

In conclusion, the current study showed that the 5-HTTLPR and gender modulate the way healthy individuals detect others' facial expressions. Low-functioning allele carriers (i.e., S/S-carriers) showed a strong positivity advantage compared with high-functioning allele carriers (i.e., L-carriers). However, this effect of the 5-HTTLPR was observed at a higher intensity of facial expressions among males than females. As the perception of positive facial expressions appears to affect our social behavior, our interactions with others could be influenced by the currently found effect of 5-HTTLPR on the detection of facial expressions. Moreover, it has been suggested that the previously demonstrated prioritized processing of negative information among low-functioning allele carriers makes them vulnerable to mood disorders [6], [15]. The current finding that the low-functioning allele enhances the processing of positive facial expressions at the perceptual detection stage may provide insights into ways of alleviating such a negativity bias and preventing the development of mood disorders. Future research should examine whether training to rely more on the perceptual experience *per se* than on the conceptual interpretation (i.e., labeling) of others' facial expressions can help ease the negativity bias and vulnerability to mood disorders among carriers of the low functioning allele of 5-HTTLPR.

## References

[1] Lesch KP, Bengel D, Heils A, Sabol SZ, Greenberg BD, et al (1996) Association of anxiety-related traits with a polymorphism in the serotonin transporter gene regulatory region. Science 274: 1527–1531. 8929413 10.1126/science.274.5292.1527

[2] Hu XZ, Lipsky RH, Zhu G, Akhtar LA, Taubman J, et al (2006) Serotonin transporter promoter gain-of-function genotypes are linked to obsessive-compulsive disorder. Am J Hum Genet 78: 815–826. 16642437 10.1086/503850 PMC1474042

[3] Pezawas L, Meyer-Lindenberg A, Drabant EM, Verchinski BA, Munoz KE, et al (2005) 5-HTTLPR polymorphism impacts human cingulate-amygdala interactions: a genetic susceptibility mechanism for depression. Nat Neurosci 8: 828–834. 15880108 10.1038/nn1463

[4] Perez-Edgar K, Bar-Haim Y, McDermott JM, Gorodetsky E, Hodgkinson CA, et al (2010) Variations in the serotonin-transporter gene are associated with attention bias patterns to positive and negative emotion faces. Biol Psychol 83: 269–271. 19723555 10.1016/j.biopsycho.2009.08.009 PMC2834856

[5] Beevers CG, Wells TT, Ellis AJ, McGeary JE (2009) Association of the serotonin transporter gene promoter region (5-HTTLPR) polymorphism with biased attention for emotional stimuli. J Abnorm Psychol 118: 670–681. 19685963 10.1037/a0016198 PMC2841741

[6] Thomason ME, Henry ML, Paul Hamilton J, Joormann J, Pine DS, et al (2010) Neural and behavioral responses to threatening emotion faces in children as a function of the short allele of the serotonin transporter gene. Biol Psychol 85: 38–44. 20493234 10.1016/j.biopsycho.2010.04.009 PMC2914171

[7] Antypa N, Cerit H, Kruijt AW, Verhoeven FE, Van der Does AJ (2011) Relationships among 5-HTT genotype, life events and gender in the recognition of facial emotions. Neuroscience 172: 303–313. 20971165 10.1016/j.neuroscience.2010.10.042

[8] Joormann J, Gotlib IH (2006) Is this happiness I see? Biases in the identification of emotional facial expressions in depression and social phobia. J Abnorm Psychol 115: 705–714. 17100528 10.1037/0021-843X.115.4.705

[9] Yoon KL, Joormann J, Gotlib IH (2009) Judging the intensity of facial expressions of emotion: depression-related biases in the processing of positive affect. J Abnorm Psychol 118: 223–228. 19222328 10.1037/a0014658 PMC2835523

[10] Lieberman MD, Eisenberger NI, Crockett MJ, Tom SM, Pfeifer JH, et al (2007) Putting feelings into words: affect labeling disrupts amygdala activity in response to affective stimuli. Psychol Sci 18: 421–428. 17576282 10.1111/j.1467-9280.2007.01916.x

[11] Friedel E, Schlagenhauf F, Sterzer P, Park SQ, Bermpohl F, et al (2009) 5-HTT genotype effect on prefrontal-amygdala coupling differs between major depression and controls. Psychopharmacology (Berl) 205: 261–271. 19387615 10.1007/s00213-009-1536-1

[12] Pacheco J, Beevers CG, Benavides C, McGeary J, Stice E, et al (2009) Frontal-limbic white matter pathway associations with the serotonin transporter gene promoter region (5-HTTLPR) polymorphism. J Neurosci 29: 6229–6233. 19439600 10.1523/JNEUROSCI.0896-09.2009 PMC2720042

[13] Stanislaw H, Todorov N (1999) Calculation of signal detection theory measures. Behav Res Methods Instrum Comput 31: 137–149. 10495845 10.3758/bf03207704

[14] Esteves F, Ohman A (1993) Masking the face: recognition of emotional facial expressions as a function of the parameters of backward masking. Scand J Psychol 34: 1–18. 8322040 10.1111/j.1467-9450.1993.tb01096.x

[15] Koizumi A, Kitagawa N, Kitamura MS, Kondo HM, Sato T, et al (2010) Serotonin transporter gene and inhibition of conflicting emotional information. Neuroreport 21: 422–426. 20216333 10.1097/WNR.0b013e32833833f0

[16] Gelernter J, Kranzler H, Cubells JF (1997) Serotonin transporter protein (SLC6A4) allele and haplotype frequencies and linkage disequilibria in African- and European-American and Japanese populations and in alcohol-dependent subjects. Hum Genet 101: 243–246. 9402979 10.1007/s004390050624

[17] Lee BT, Ham BJ (2008) Serotonergic genes and amygdala activity in response to negative affective facial stimuli in Korean women. Genes Brain Behav 7: 899–905. 18826444 10.1111/j.1601-183X.2008.00429.x

[18] Murakami F, Shimomura T, Kotani K, Ikawa S, Nanba E, et al (1999) Anxiety traits associated with a polymorphism in the serotonin transporter gene regulatory region in the Japanese. J Hum Genet 44: 15–17. 9929970 10.1007/s100380050098

[19] Li S, Zou Q, Li J, Li J, Wang D, et al (2012) 5-HTTLPR polymorphism impacts task-evoked and resting-state activities of the amygdala in Han Chinese. PLoS One 7: e36513. 22574175 10.1371/journal.pone.0036513 PMC3344896

[20] Chiao JY, Blizinsky KD (2010) Culture-gene coevolution of individualism-collectivism and the serotonin transporter gene. Proc Biol Sci 277: 529–537. 19864286 10.1098/rspb.2009.1650 PMC2842692

[21] Hariri AR, Mattay VS, Tessitore A, Kolachana B, Fera F, et al (2002) Serotonin transporter genetic variation and the response of the human amygdala. Science 297: 400–403. 12130784 10.1126/science.1071829

[22] Hoffmann H, Kessler H, Eppel T, Rukavina S, Traue HC (2010) Expression intensity, gender and facial emotion recognition: Women recognize only subtle facial emotions better than men. Acta Psychol (Amst) 135: 278–283. 20728864 10.1016/j.actpsy.2010.07.012

[23] Hall JA, Matsumoto D (2004) Gender differences in judgments of multiple emotions from facial expressions. Emotion 4: 201–206. 15222856 10.1037/1528-3542.4.2.201

[24] Beck AT, Ward CH, Mendelson M, Mock J, Erbaugh J (1961) An inventory for measuring depression. Arch Gen Psychiatry 4: 561–571. 13688369 10.1001/archpsyc.1961.01710120031004

[25] Ekman P, Friesen W (1978) Facial action coding system: A technique for the measurement of facial movement. Consulting Psychologists Press: Palo Alto

[26] Brainard DH (1997) The Psychophysics Toolbox. Spat Vis 10: 433–436. 9176952

[27] Summerfield C, Egner T, Greene M, Koechlin E, Mangels J, et al (2006) Predictive codes for forthcoming perception in the frontal cortex. Science 314: 1311–1314. 17124325 10.1126/science.1132028

[28] Gorea A, Sagi D (2000) Failure to handle more than one internal representation in visual detection tasks. Proc Natl Acad Sci USA 97: 12380–12384. 11050253 10.1073/pnas.97.22.12380 PMC17350

[29] Green DM, Swets JA (1966) Signal detection theory and psychophysics. New York: Wiley.

[30] Macmillan NA (1993) Signal detection theory as data analysis method and psychological decision model. In: G. Karen CL, editor. A handbook for data analysis in the behavioral sciences: Methodological issues. Hillsdale: Erlbaum. pp. 21–57.

[31] Lee E, Kang JI, Park IH, Kim JJ, An SK (2008) Is a neutral face really evaluated as being emotionally neutral? Psychiatry Res 157: 77–85. 17804083 10.1016/j.psychres.2007.02.005

[32] Becker DV, Neel R, Srinivasan N, Neufeld S, Kumar D, et al (2012) The vividness of happiness in dynamic facial displays of emotion. PLoS One 7(1): e26551. 22247755 10.1371/journal.pone.0026551 PMC3256131

[33] Beevers CG, Marti CN, Lee HJ, Stote DL, Ferrell RE, et al (2011) Associations between serotonin transporter gene promoter region (5-HTTLPR) polymorphism and gaze bias for emotional information. J Abnorm Psychol 120: 187–197. 21319930 10.1037/a0022125

[34] Bishop S, Duncan J, Brett M, Lawrence AD (2004) Prefrontal cortical function and anxiety: controlling attention to threat-related stimuli. Nat Neurosci 7: 184–188. 14703573 10.1038/nn1173

[35] Caspi A, Sugden K, Moffitt TE, Taylor A, Craig IW, et al (2003) Influence of life stress on depression: moderation by a polymorphism in the 5-HTT gene. Science 301: 386–389. 12869766 10.1126/science.1083968

